# Influence of Cutting the Hair on Health

**Published:** 1849-07

**Authors:** 


### ARTICLE VI.

*Influence of Cutting the Hair on Health.* By Dr. Fredericq.

A beautiful child, æt. 3 years, had long hair descending to her shoulders. But she for some time lost flesh and became dull, lost her color and appetite. No organic lesion was discoverable but a souffle was heard in the carotids. Chalybeates failed to improve her condition. Her beautiful hair was then sacrificed, and her health was soon after restored.

The supply of coloring matter to so much of it had exhausted the hæmatosine of the blood. Hair being badly nourished, often falls after illness, and is not always reproduced. It is then advisable to cut short the hair of the convalescents.

[ In females it is quite common to meet with this result The editor never remembers to have witnessed its occurence in a male. The practice suggested is generally adopted, but not on the scientific principle here set forth. Ed.]
—*Flanders Journal.*

